# Tetraploidy in *Citrus wilsonii* Enhances Drought Tolerance *via* Synergistic Regulation of Photosynthesis, Phosphorylation, and Hormonal Changes

**DOI:** 10.3389/fpls.2022.875011

**Published:** 2022-04-28

**Authors:** Jinglong Jiang, Ni Yang, Li Li, Gongwei Qin, Kexin Ren, Haotian Wang, Jiarui Deng, Dekuan Ding

**Affiliations:** ^1^School of Biological Science and Engineering, Shaanxi University of Technology, Hanzhong, China; ^2^Chenggu Fruit Industry Technical Guidance Station, Chenggu, China

**Keywords:** tetraploid, drought, phytohormones, photosynthesis, phosphorylation

## Abstract

Polyploidy varieties have been reported to exhibit higher stress tolerance relative to their diploid relatives, however, the underlying molecular and physiological mechanisms remain poorly understood. In this study, a batch of autotetraploid *Citrus wilsonii* were identified from a natural seedling population, and these tetraploid seedlings exhibited greater tolerance to drought stress than their diploids siblings. A global transcriptome analysis revealed that a large number of genes involved in photosynthesis response were enriched in tetraploids under drought stress, which was consistent with the changes in photosynthetic indices including P_n_, gs, T_r_, C_i_, and chlorophyll contents. Compared with diploids, phosphorylation was also modified in the tetraploids after drought stress, as detected through tandem mass tag (TMT)-labeled proteomics. Additionally, tetraploids prioritized the regulation of plant hormone signal transduction at the transcriptional level after drought stress, which was also demonstrated by increased levels of IAA, ABA, and SA and reduced levels of GA3 and JA. Collectively, our results confirmed that the synergistic regulation of photosynthesis response, phosphorylation modification and plant hormone signaling resulted in drought tolerance of autotetraploid *C. wilsonii* germplasm.

## Introduction

*Citrus wilsonii* is a citrus rootstock native to the Southern Qinling Mountains in China that has been cultivated for more than 500 years (Li et al., 2018). The rootstock cultivar of *C. wilsonii* is also called “Zhique” in Chenggu county of Shaanxi province, China, where it has been cultivated as a traditional medicine in the Chinese Pharmacopoeia (Cheng et al., 2017). Zhique is a natural hybrid citrus variety with pummelo (*Citrus maxima*) as the female parent and trifoliate orange [*Poncirus trifoliata* (L.) Raf.] as the male parent, based on nuclear and chloroplast simple sequence repeat (SSR) markers analysis (Li et al., 2018). As a citrus rootstock, Zhique shows strong tolerance to iron deficiency in calcareous soil that is better than the most commonly used trifoliate orange rootstock (Fu et al., 2016). Further studies demonstrated that tolerance to iron deficiency in Zhique rootstocks could be due to higher iron uptake rate and increased iron translocation from roots to shoots, and that cell wall modification and ethylene and abscisic acid (ABA) signaling pathways seem to be involved (Fu et al., 2017). Therefore, Zhique accessions have great potential as a stress resistant rootstock for grafting citrus.

Polyploidy, a state in which two or more complete sets of chromosomes co-occur in a cell, is a major force in plant evolution (Ramsey and Schemske, 2002). Polyploids may be allotetraploids or autotetraploids, resulting either from sexual reproduction *via* 2n gametes or somatic chromosome doubling, respectively (Allario et al., 2013). Autopolyploid genomes are composed of identical sets of chromosomes, while allotetraploid genomes are composed of distinguishable subgenomes (Soltis and Soltis, 2009). As previously reported, autotetraploidization has profound effects on the morphology and physiology of citrus, including shorter plant height, larger organ size, and different anatomical features (Tan et al., 2019). Despite the fact that citrus can produce tetraploid seedlings at relatively high frequency, tetraploidy seems to have played a negligible part in the evolution of citrus fruits (Ollitrault et al., 2008). Autopolyploid (also named double diploid) citrus rootstocks have been shown to be more tolerant to abiotic stresses, such as drought (Allario et al., 2013; Wei et al., 2019), chilling (Oustric et al., 2017), heat (Zhang et al., 2010), salt (Tu et al., 2014; Ruiz et al., 2016; Liu and Sun, 2017), and chromium toxicity (Balal et al., 2017), than their diploid relatives. Citrus autopolyploids have been receiving increased attention due to their tolerance to abiotic stresses compared to their diploid progenitors, however, there is a paucity of knowledge concerning the molecular events associated with stress responses in the autotetraploids.

Plants constantly receive various challenges from a myriad of biotic and abiotic stresses during their life span. Among the abiotic stresses, drought stress is one of the main constraints to agricultural development and productivity (Li et al., 2019; Geng et al., 2020). In fruit trees, water deficit not only affects the growth and development of the perennial tree, but also inhibits flower bud differentiation and annual vegetative growth, thereby causing flowers and fruits to drop (Virlet et al., 2015; Niu et al., 2019). In China, most citrus cultivation occurs in mountainous areas, where water is scarce. Therefore, there is a push to find naturally drought-resistant plants to produce stress-tolerant germplasms. Polyploids display various responses to drought stress relative to their diploid progenitors, thus it is proposed that tetraploid citrus germplasm may hold great potential for improving drought resistance. For example, rootstock of tetraploid Rangpur lime (*Citrus limonia*) shows increased drought tolerance compared to its diploid progenitors *via* enhanced constitutive ABA production in the root (Allario et al., 2013). Wei et al. (2019) also reported that autotetraploid trifoliate orange exhibits more tolerance to drought and dehydration stress than related diploid plants because of enhanced reactive oxygen species (ROS) scavenging and sugar accumulation.

In this study, we obtained naturally occurring tetraploids among Zhique seedlings and found that they exhibited more tolerance to drought stress than the diploid seedlings. These *C. wilsonii* autotetraploids will be tested as germplasm to improve stress tolerance for grafting citrus. Under drought stress, the tetraploid plants exhibited fewer differentially expressed genes (DEGs) than the diploid in a global RNA sequencing analysis. The predominant DEGs enriched in the tetraploid were involved in photosynthesis, which was consistent with the results of the measured photosynthetic indices. However, more DEGs involved in carbohydrate metabolic process were enriched in the diploid. Further studies indicated that phosphorylation modification and plant hormone signal regulation also played important roles in the tetraploid plants relative to the diploid counterpart.

## Materials and Methods

### Plant Culture and Autotetraploid Seedlings Screen

*Citrus wilsonii* (“Zhique” in Chinese) fruits were harvested from trees grown in the Citrus Germplasm Repository of Chenggu Fruit Industry Technical Guidance Station, (33°04′N, 107°02′E, Hanzhong, Shaanxi, China) (Supplementary Figure 1). Sterilized seeds were germinated in plastic plug trays (50 cm in length, 26 cm in width, and 4.5 cm in height) filled with commercial soil in the greenhouse. When the seedlings reached the three-leaf stage, seedlings were transplanted into perlite-filled plastic pots (11.5-cm diameter and 13-cm height).

Four-month-old seedlings were screened for potential tetraploidy according to morphology features, especially thicker and shorter roots, based on the methods of Aleza et al. (2011) and Tan et al. (2015). The candidate tetraploids were subjected to flow cytometry (FCM) to determine their ploidy levels (Wei et al., 2019) and were observed microscopically to count their chromosomes in the root tip (Gunasekaran et al., 2019). Finally, autotetraploid seedlings were identified by SSR genetic analysis with three pairs of primers (Supplementary Table 1; Barkley et al., 2006).

### Morphological and Microscopic Observation

The autotetraploid and counterpart diploid seedlings, grown under the same conditions for four months, were assessed for morphological indices including plant height, leaf size, the densities of oil glands and stomata, and microscopic observation. Ten independent diploid or tetraploid plants assessed for plant height and root diameter. Fully expended leaves from ten independent diploid or tetraploid plants were collected to measure the leaf width, leaf thickness, density of oil glands, and stomatal density. The stomatal density and size were observed by scanning electron microscopy (S-3400N; Hitachi, Tokyo, Japan) (Morillon and Chrispeels, 2001). The density of oil glands was counted within a 1-cm^2^ field of view each time. Longitudinal cross-sections of paraffin-embedded leaves were prepared according to the description of Allario et al. (2011). Leaf samples were fixed and embedded in Micro-Bed resin (Electron Microscopy Sciences, Fort Washington, PA, United States) (Tadeo et al., 1995). Sections (about 1 mm thick) were cut with a Leica RM2255 microtome (Leica Microsystems, Wetzlar, Germany) using glass knives and fixed to microscope slides. Cross-sections of leaf were stained with 0.05% Toluidine Blue O (CI 52040; Merck, Darmstadt, Germany) and then examined and photographed with a Leica DM LA microscope (Leica Microsystems, Wetzlar, Germany). The morphometrical analysis was performed on highly contrasted micrographs with the Leica IM software (Leica Microsystems, Wetzlar, Germany).

### Drought Stress Treatments and Sampling

Ten-month-old autotetraploid and counterpart diploid plants were chosen for drought stress treatment by withholding irrigation for 21 days. The 10-month-old plants employed are not subjected to grafting during the whole experimental process. The drought stressed and the control groups each contained six independent tetraploid and diploid plants. Plants in the control groups were irrigated with 40 mL deionized water every 3 days at 9:00 a.m. during the whole treatment process. The seedlings were maintained in a controlled growth chamber with a 16-h light/8 h dark regime, a relative humidity of 80%, a temperature of 25°C and a photosynthetically active radiation of 800 μmol m^–2^ s^–1^. It is worth noting that there exists a significant difference in the growth status of seedlings cultivated in the open field compared to those in enclosed cultivation rooms. After drought stress treatment, two fully expanded leaves (the third and fourth from the top) were selected from each plant to determine the *in vivo* chlorophyll fluorescence [the ratio of variable (F_v_) to maximum fluorescence (F_m_)] and photosynthesis parameters, including net photosynthetic rate (P_n_), transpiration rate (T_r_), stomatal conductance (gs), and intercellular CO_2_ concentration (C_i_). The fresh leaves from two plants were pooled as an independent biological replicate, and three independent biological replicates were analyzed for each sample. These fresh leaves were harvested to assess the electrolyte leakage, relative water content (RWC) and leaf water potential. Other leaves were sampled and immediately frozen in liquid nitrogen and then stored at −80°C until RNA extraction [for sequencing or quantitative real-time PCR (qRT-PCR) analysis], phosphoproteomic analyses, plant hormone determination or other physiological analyses such as the determination of the levels of chlorophyll *a*, chlorophyll *b*, carotenoids, glucose, fructose, and soluble starch.

### Physiological Index Measurements

Soil moisture content and RWC of leaf and root were measured (Barr and Weatherly, 1962). Membrane integrity was evaluated in the leaves by measuring electrolyte leakage (Jiang et al., 2019a). The leaf water potential was measured according to the method of Bonal and Guehl (2011). The contents of glucose, fructose and soluble starch were determined by anthrone colorimetry (Zhang, 1977). The experiments were performed with three independent biological replicates.

Chlorophyll fluorescence is considered a tool for interpreting the stress tolerance of plants by evaluating the physiological status of the plant and the state of Photosystem II. The ratio of variable (F_v_) to maximum fluorescence (F_m_) was measured using a portable chlorophyll fluorometer (OS-30p^+^, Opti-Science, Inc., Tyngsboro, MA, United States). The leaves were dark adapted for more than 30 min prior to the measurement. The minimum fluorescence (F_o_), the F_m_, the variable fluorescence (F_v_ = F_m_−F_o_) and the ratio of F_v_/F_m_ were recorded for 15 s at a 100% intensity level of the photon flux density (600 μmol m^–2^ s^–1^) according to the methods of Hu and Yu (2014). The photosynthesis indices of fully expanded leaves, including P_n_, T_r_, gs, and Ci, were determined as described by Chen et al. (2016) using a portable photosynthesis system (Li-6400, Li-Cor, Lincoln, NE, United States). Gas exchange was measured at PAR levels of 600 μmol m^–2^ s^–1^. All measurements were conducted in the morning (9:00–11:00) to avoid the high temperature and air vapor pressure deficits in the afternoon. Light was supplemented using a light emitting diode light system. Using non-rectangular hyperbola modeling, the response of leaf P_n_ to PAR was calculated with respect to the apparent dark respiration (Rd), light compensation point (Lcp), light saturation point (Lsp), apparent quantum yield (AQE), and maximal net photosynthetic rate (Pmax), as described by Prioul and Chartier (1977).

The contents of Chlorophyll *a*, chlorophyll *b*, and carotenoids were measured on fresh leaves as described by Lichtenthaler and Wellburn (1983). The first true leaf was chosen to determine the parameters of photosynthesis, chlorophyll fluorescence and chlorophyll content. Three independent biological replicates were conducted per treatment under the same experimental conditions.

### RNA Sequencing and Transcriptome Analysis

The leaves of the diploids and the tetraploids under well-watered or drought conditions were harvested for transcriptome analysis. Leaves from two plants were pooled as an independent biological replicate, and three independent biological replicates were analyzed for each sample. Total RNA was isolated from each treatment using the RNAplant Plus Reagent DP437 (Tiangen, Beijing, China). The concentration and quality of RNA were determined using an Agilent 2100 Bioanalyzer (Agilent Technologies, Santa Clara, CA, United States). A total of 20 mg of RNA from each sample was used for cDNA library construction. The library products were ready for sequencing analysis *via* Illumina HiSeq™ 2000 (Illumina, San Diego, CA, United States), which was performed at the Shanghai Personal Biotechnology Co. Ltd. for RNA-Seq analysis (Personalbio, Shanghai, China). The RNA-Seq raw sequencing data was filtered using the Cutadapt program.^1^ The filtered clean reads were further mapped to the reference genome (HWB.chromosome.fa) and downloaded from the database^2^ using Tophat2^3^ with up to two mismatches allowed. The transcripts were screened for significant changes in abundance using DESeq^4^ with a condition of | log_2_ fold-change| > 1.0 and *p*-value < 0.05. Principal component analysis (PCA) was performed on distance matrices, and coordinates were used to draw 2D graphical outputs (Lozupone et al., 2007; Jiang et al., 2019b). Gene Ontology (GO) annotation was derived from the GO knowledgebase.^5^ All DEGs were searched against the Kyoto Encyclopedia of Genes and Genomes (KEGG) database (KEGG^6^) to identify the main metabolic pathways and signal transduction pathways of DEGs using Blastall software (Kanehisa and Goto, 2000). Hierarchical clusters were assembled using the complete linkage clustering method through MeV software according to Jiang et al. (2020).

### Real-Time Quantitative Reverse Transcription PCR and Pearson Correlation Analysis

The total RNA samples were treated with RNAase-free DNase (TaKaRa Bio Inc., Dalian, China) to eliminate traces of DNA, followed by quantification using a NanoDrop 2000 (Thermo Fisher Scientific, Wilmington, DE, United States). Total RNA (2 μg) was reverse-transcribed using an oligod(T) primer (50 μM, 1 μL) and M-MLV reverse transcriptase (200 U/μL, 1 μL) (BioTeke, Beijing, China). Real-time quantitative RT-PCR reactions were performed using an ABI 7000 (Applied Biosystems) with SYBR^®^Premix ExTaq™ (TaKaRa Bio Inc., China) and the cycling conditions of denaturation at 95°C for 5 min, followed by 40 cycles of denaturation at 95°C for 15 s, annealing at 60°C for 30 s, and extension at 72°C for 15 s. Using specific primers (Supplementary Table 2), the expression levels of the genes were presented as values relative to the corresponding control samples under the indicated conditions, with normalization of data to the geometric average of the internal control gene *actin*. Three independent replicates were performed for each sample. The comparative threshold cycle (Ct) method was used to determine the relative amount of gene expression. Relative gene expression levels were calculated *via* the 2^–ΔΔ*CT*^ method (Livak and Schmittgen, 2001). Correlation of expression between RNA-seq (*Y*-axis) and RT-qPCR (*X*-axis) were analyzed using the Pearson correlation analysis.

### Phosphoproteomic Analyses

Frozen leaf tissue (100 mg) of the tetraploid plants with or without drought stress was used for phosphoproteomic analyses. Three parallel replicates were set up for the control group (TC) or drought stress group (TD). Phosphopeptides were enriched using immobilized metal-ion affinity chromatography (IMAC) resin (PHOS-Select™ Iron Affinity Gel, Sigma-Aldrich) (Bigeard et al., 2014). Proteomic analysis, including protein preparation, trypsin digestion, tandem mass tag (TMT) labeling, and liquid chromatography (LC)–mass spectrometry (MS)/mass spectrometry (MS) analysis was performed according to the detailed description of Jiang et al. (2021). The entire phosphoproteomic analysis was done at PTM BIO Company (Hangzhou, China).

### Plant Hormone Determination

Frozen leaf tissue (100 mg) was extracted in 1 mL of ice-cold 50% aqueous (v/v) acetonitrile, and then extracted using a Stuart SB3 benchtop laboratory rotator (Bibby Scientific) for 4 h at 4°C. After centrifugation for 10 min at 36, 670 × *g* at 4°C, the supernatant was transferred to clean plastic microtubes. All samples were purified using a Max cartridge that had been washed with 4 mL of 100% methanol and 2 mL 0.1 M ammonia solution. After the sample was concentrated under vacuum and 0.1 M aqueous ammonia solution was added to bring the volume to 2 mL, it was passed through a MAX cartridge. Each sample was washed with 2 mL of 0.1 M ammonia water solution and then 2 mL of 0.1 M ammonia water and 60% methanol solution. Finally, the sample was dissolved by adding 0.2 ml methanol and stored at −20°C until analysis.

Targeted compounds were analyzed using an UPLC System (Waters, Milford, MA, United States) equipped with a Waters HSS T3 liquid chromatography column (50 × 2.1 mm, 1.8 μm I.D.), The injection volume was 2 μL, column temperature was 40°C, mobile phase A was 0.1% acetonitrile, and mobile phase B was 0.1% acetic acid/water. The calibration curves of UPLC determination are shown in Supplementary Table 3. The original data for all quality control (QC) samples were collected for data pre-treatment, including peak extraction, arrangement and normalization, and the relative standard deviation (RSD) value of the peak area was calculated. The quality control (QC) showed that the RSD for each hormone was less than 5%, indicating that the quality of the detection data is reliable and can be used for subsequent analysis (Supplementary Table 4). The raw UHPLC-ESI-MS/MS data is shown in Supplementary Table 5. MS/MS analysis was performed on a mass spectrometry system (Q Exactive, Thermo, United States) equipped with an electrospray ionization (ESI) source (AB SCIEX, Foster City, CA, United States) and XCALIBUR workstation. To improve sensitivity, the sample was analyzed in single ion detection (SIM) mode under negative ions. The optimized ESI operating parameters for negative mode were: sleek, 40; auxiliary gas, 10; ion spray voltage, −2,800 V; temperature, 350°C; and ion transmission tube temperature, 320°C.

### Statistical Analysis

Statistical analysis of the data, shown as means ± SE, was conducted using one-way analysis of variance (ANOVA) and *t*-test in SPSS (IBM, New York, NY, United States).

## Results

### Screening and Molecular Identification of Autotetraploids in *Citrus wilsonii*

**FIGURE 1:**
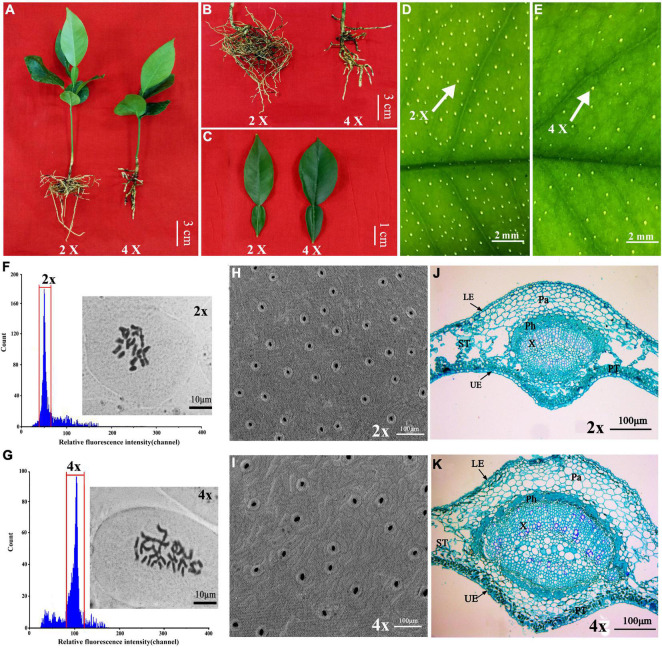
Morphological and microscopic comparison between tetraploid and diploid in *Citrus wilsonii*. Plant height and morphology in 4-month-old diploid and tetraploids **(A)**; roots morphology **(B)**; leaf morphology **(C)**; the density of oil glands cell in diploid **(D)** and tetraploid **(E)** leaves; ploidy level of diploid **(F)** and tetraploid **(G)** plants using FCM (flow cytometry) and chromosome counting analysis; scanning electronic microscopic observation of stomata in diploid **(H)** and tetraploid **(I)** leaves; microscopic observation using cross sections of diploid **(J)** and tetraploid **(K)** leaves. UE, upper epidermal; LE, lower epidermal; ST, spongy tissue; PT, palisade tissue; Ph, phloem; X, xylem; Pa, parenchyma tissue.

In a population of 570 Zhique plants germinated from seeds, 21 plants were preliminarily identified during transplanting as potential tetraploids according to morphological characteristics such as dwarf stature (Figure 1A), stubby roots (Figure 1B), thicker and wider leaves (Figure 1C), and lower density of oil gland cells on the surface of leaves (Figures 1D,E). The ploidy levels of these candidate tetraploids were subsequently determined *via* both FCM and chromosome counting in the root tip. In FCM, the fluorescence intensity of the diploid (2×) cells peaked at about 50 (Figure 1F), while that of the tetraploid (4×) peaked at 100 (Figure 1G). The diploid (2×) plants contained 18 chromosomes (Figure 1F), while the tetraploid (4×) plants had 36 chromosomes (Figure 1G). Finally, 17 plants were identified as tetraploids (4×). The rate of preliminary screening was 80.95% and of population natural incidence was 2.98%. The tetraploid plants had the same SSR molecular marker pattern as their corresponding diploid parents, showing a highly consistent genetic background and proving that the tetraploid genotype is an autotetraploid stemming from doubling of the chromosome set in somatic cells of the diploid parent (Supplementary Figure 2).

There were significant differences between the diploid and tetraploid plants under the same growth conditions. The tetraploid plants exhibited slower growth, shorter stature, more sturdy roots, and thicker and wider leaves compared to the diploid plants (Figures 1A–C). The plant height, root diameter and leaf thickness and width of tetraploid plants were 71.24%, 6.5-fold, 1.15-fold, and 1.42-fold of the diploids, respectively (Table 1). Additionally, tetraploid plants had a lower stomatal density (166.88 per mm^2^) on the surface of leaves than the diploid plants (304.00 per mm^2^) (Figures 1H,I). The density of oil gland cells was 170.80 per mm^2^ in the diploid plants, but only 52.34 per mm^2^ in the tetraploid plants (Table 1). Microscopic observation showed that the leaf blade was significantly thicker in the tetraploid plants because of larger palisade parenchyma, lacunar parenchyma, and epidermal cells (Figures 1J,K). These observations clearly indicated that the tetraploids were morphologically and anatomically different from their diploid siblings.

**TABLE 1:** Morphology and stomatal indices of tetraploid and diploid seedlings of *Citrus wilsonii*.

Index	Plant height (cm) (*n* = 10)	Root diameter (cm) (*n* = 10)	Leaf width (cm) (*n* = 10)	Leaf thickness (μm) (*n* = 10)	Oil gland density (N/cm^2^) (*n* = 10)	Stomata length (μm) (*n* = 10)	Stomata width (μm) (*n* = 10)	Stomata density (N/mm^2^) (*n* = 10)
Diploid	12.76 ± 0.45**	0.12 ± 0.01	2.45 ± 0.20	316.68 ± 8.94	170.80 ± 35.08	19.43 ± 0.40	15.50 ± 0.36	304.00 ± 4.87
Tetraploid	9.09 ± 0.15	0.78 ± 0.01**	3.47 ± 0.12**	365.17 ± 5.02*	52.34 ± 15.6*	27.40 ± 0.46**	21.63 ± 0.50*	166.88 ± 4.87**

Statistical analysis of the data, shown as means ± SE, was conducted using t-test analysis in SPSS (IBM, New York, NY, United States), taking *P < 0.05, **P < 0.01 as significance. N: number and n: repeated number of samples.

### Tetraploid Zhique Exhibited Enhanced Drought Tolerance

**FIGURE 2:**
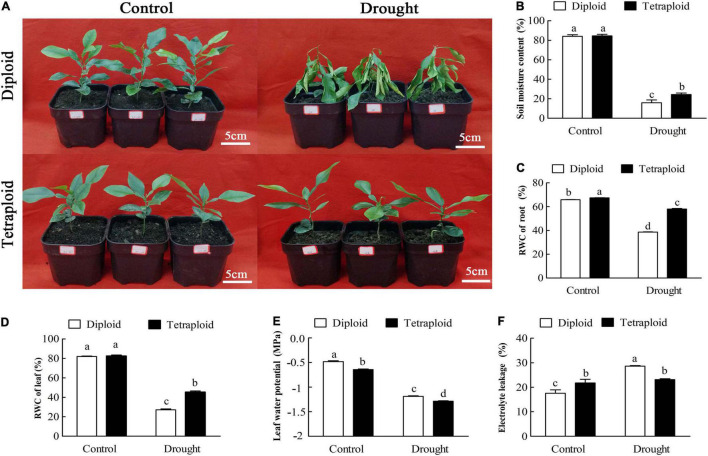
Morphological and physiological analysis between tetraploids and diploids *Citrus wilsonii* in response to drought stress. The morphological changes of tetraploids and diploids *Citrus wilsonii* under drought stress **(A)**; physiological analysis in soil moisture content **(B)**, RWC of root **(C)**, RWC of leaf **(D)**, leaf water potential **(E)**, and electrolyte leakage **(F)** between tetraploids and diploids under drought stress. Each value is the mean of three biological replicates, and the vertical bars represent the standard errors. Values sharing the same lower case letters are not significant by ANOVA at *P* < 0.05.

Ten-month-old diploid and tetraploid plants were subjected to drought stress by withholding watering for 21 days. The control group was irrigated with deionized water every 3 days. After 21 days of drought stress, the diploid plants began to show the effects of severe water loss, leaf wilting and even lodging, compared with plants in the control group (Figure 2A). On the other hand, the tetraploid plants did not show the above effects, indicating that they exhibited greater drought resistance relative to the diploids (Figure 2A). When drought stress was prolonged to 32 days, the tetraploid plants began to show symptoms of dehydration and wilting (data not shown). Compared to the watered control, the diploid plants showed significant (*p* ≤ *0.05*) decreases in soil moisture content (by 5.28-fold), RWC of root (by 1.71-fold), RWC of leaf (by 1.72-fold) and leaf water potential (by 2.48-fold) and a significant (*p* ≤ *0.05*) increase in electrolyte leakage (1.63-fold higher than control) when under drought stress. However, the tetraploids exhibited smaller decreases in soil moisture content (by 3.46-fold), RWC of root (by 1.16-fold) and of leaf (by 1.39-fold), and leaf water potential (by 2.01-fold) and increase in electrolyte leakage (by 1.06-fold) relative to the control under drought stress (Figures 2B–F). The data above indicated that the tetraploids displayed less water loss and cellular damage than the diploids when water was withheld, which indicated that the tetraploids had more tolerance to drought stress.

### Transcriptional Profiling of Tetraploids and Diploids Under Drought Stress

To begin to understand the molecular mechanisms underlying the enhanced drought tolerance of tetraploid Zhique plants, transcriptome profiles of leaves were generated using RNA-seq. The well-watered diploid and tetraploid control plants were designated as DC and TC, respectively, and the drought-stressed diploid and tetraploid plants were designated as DD and TD, respectively. After filtering raw reads, a total of 3,548,821,144 clean reads were obtained from the twelve samples, accounting for 91.5% of the total reads (Q_30_ > 93.0%) (Supplementary Table 6). The clean reads mapped to the pummelo reference genome with a 91.0% total mapping ratio, comprised of 88.1% single matches and 2.95% multiple gene matches (Supplementary Table 7).

**FIGURE 3:**
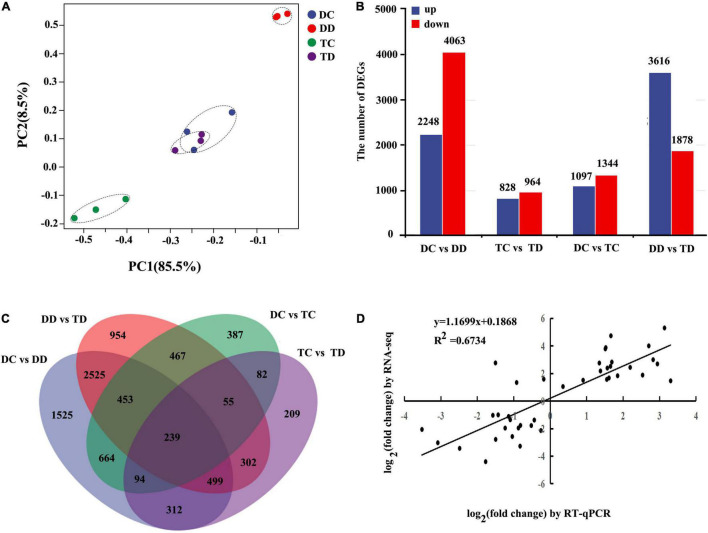
Differentially expressed genes in diploids and tetraploids *Citrus wilsonii* with or without drought treatment were analyzed by RNA-seq and RT-qPCR. The similarity of the expression of genes was compared between sample groups using PCA. The top 500 genes with the highest contribution were chosen for PCA analysis **(A)**; comparison analysis of DEGs in the DD vs TD, TC vs TD, DC vs TC, and DC vs DD were shown in column chart **(B)**; common and specific DEGs were compared between the different pairwise comparisons using the Venn diagram **(C)**; correlation of expression analysis by RNA-seq (*Y*-axis) and RT-qPCR (*X*-axis) **(D)**.

The similarities of the expression values for all genes in the samples of DC, DD, TC, and TD were determined by PCA (Figure 3A). The samples of DC and DD as well as TC and TD were clearly distinct, with PC1 accounting for 85.5% of the variance. This indicated that drought stress caused obvious changes in both diploid and tetraploid transcriptomes compared with the well-watered groups. Interestingly, the DC and TD samples grouped near each other, indicating that drought-stressed tetraploid plants maintained a transcriptional profile similar to well-watered diploid plants.

The transcripts were subjected to pairwise comparisons, DC vs DD, TC vs TD, DC vs TC, and DD vs TD, using DESeq. Transcripts with a *P* < 0.05, and | log_2_ fold change| > 1 were defined as DEGs (Supplementary Table 8). A total of 16,038 (7,789 upregulated and 8,249 down-regulated) DEGs were detected from these pairwise comparisons. A total of 6,311 genes showed differential expression in the leaves of the diploid with drought stress compared to the control (DC vs DD), whereas only 1,792 DEGs were detected in the tetraploid leaves under drought stress (TC vs TD, Figure 3B), indicating that transcript levels in the diploid plants changed drastically under drought, while the transcript levels in the tetraploid plants were more stable. Additionally, there were 5,494 DEGs between the diploid and tetraploid under drought stress (DD vs TD), while there were only 2,441 DEGs between the well-watered diploid and tetraploid plants (Figure 3B). A Venn diagram of the DEGs shows how many genes are specific to one treatment and how many genes transcriptionally respond to multiple treatments (Figure 3C). Only 239 DEGs were shared in all the samples.

To further assess the reliability of the RNA-seq data, qPCR was performed to analyze the expression patterns of ten randomly selected genes. The relative expression level of each gene is shown in Supplementary Figure 3. The correlation coefficient (*R*^2^ = 0.6734) between the qPCR and RNA-seq results was high, implying that the RNA-seq data are reliable (Figure 3D). These results indicated that, under drought stress, tetraploid plants showed a relatively moderate change in global transcriptome compared to the diploid plants.

### Gene Ontology Annotation of the Differentially Expressed Genes

**FIGURE 4:**
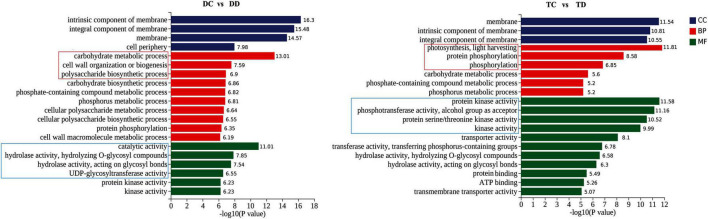
Comparative GO (Gene Ontology) in DC vs DD and TC vs TD enrichment analysis of enriched DEGs. The results are summarized in three main categories: biological process (BP), cellular component (CC), and molecular function (MF).

To delineate the differences in the enrichment of DEGs between the tetraploid and diploid plants, GO analysis of the DEGs was carried out based on three major categories, cellular component, molecular function, and biological process. As shown in Figure 4, GO terms in the cellular component category were similarly enriched in the tetraploid and diploid plants after drought stress. However, there were obvious differences in the biological process category between the diploids and the tetraploids. The top three GO terms in the tetraploids were “photosynthesis, light harvesting,” “protein phosphorylation,” and “phosphorylation,” in that order. In the diploids, the top three GO terms were “carbohydrate metabolic process,” “cell wall organization or biogenesis,” and “polysaccharide biosynthetic process” (Figure 4). In the “molecular function” category, the most-enriched GO term in the tetraploids was “protein kinase activity,” while “catalytic activity” was the top in the diploids. This indicated that the tetraploid citrus plants could switch on signal transduction by activating protein kinase activity, while the diploids altered carbohydrate metabolism. In total, these data demonstrated that photosynthesis and signal transduction (especially phosphorylation) play important roles in the tetraploid plants under drought stress, while regulating carbohydrate metabolism was still important in the diploids under drought stress.

**FIGURE 5:**
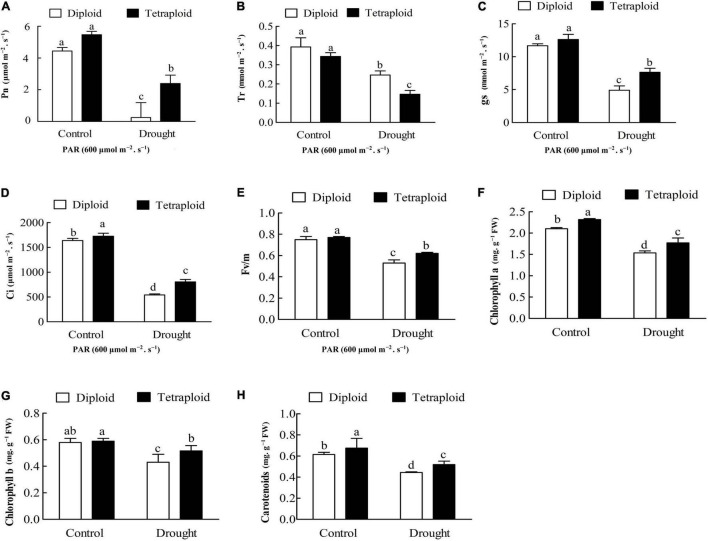
Photosynthetic analysis in diploids and tetraploids *Citrus wilsonii* under drought stress. **(A)** Pn; **(B)** Tr; **(C)** gs; **(D)** Ci; **(E)** Fv/Fm; **(F)** chlorophyll *a* content; **(G)** chlorophyll *b* content; **(H)** carotenoid content. Values sharing the same lower case letters (a–d) are not significant by ANOVA at *P* < 0.05.

Drought stress significantly (*P* < *0.05*) reduced the photosynthetic indices, including P_n_, G_S_, T_r_, C_i_, F_v_/F_m_, and chlorophyll *a* and *b* contents, in both diploid and tetraploid leaves compared with the control (Figure 5), indicating that drought stress inhibited photosynthesis. The drought-stressed diploid plants showed significant decreases for five indices, in P_n_ by 82.40%, T_r_ by 37.27%, G_S_ by 58.01%, C_i_ by 70.44%, and F_v_/F_m_ by, 29.33% compared with plants in the control group, while the drought-stressed tetraploid plants showed lesser declines for four indices (P_n_, G_S_, C_i_, and F_v_/F_m_ by 60.67, 39.54, 46.43, and 19.48%) but a greater decline for T_r_, by 57.27% (Figures 5A–E). Compared with the control, drought stress reduced chlorophyll *a* and *b* and carotenoids content by 57.85, 58.25, and 52.69% in the diploid plants, respectively, while in the tetraploid plants, the chlorophyll *a*, *b* and carotenoids content decreased by 42.80, 43.57, and 35.26% under drought stress, respectively (Figures 5F–H). The data above indicated that the tetraploid plants displayed smaller declines in the photosynthetic indices relative to the diploid plants when they were under drought stress. These differences indicated that the tetraploid Zhique rootstock maintains higher photosynthetic activities than the diploids after drought stress, which was in line with the GO analysis of the DEGs.

**FIGURE 6:**
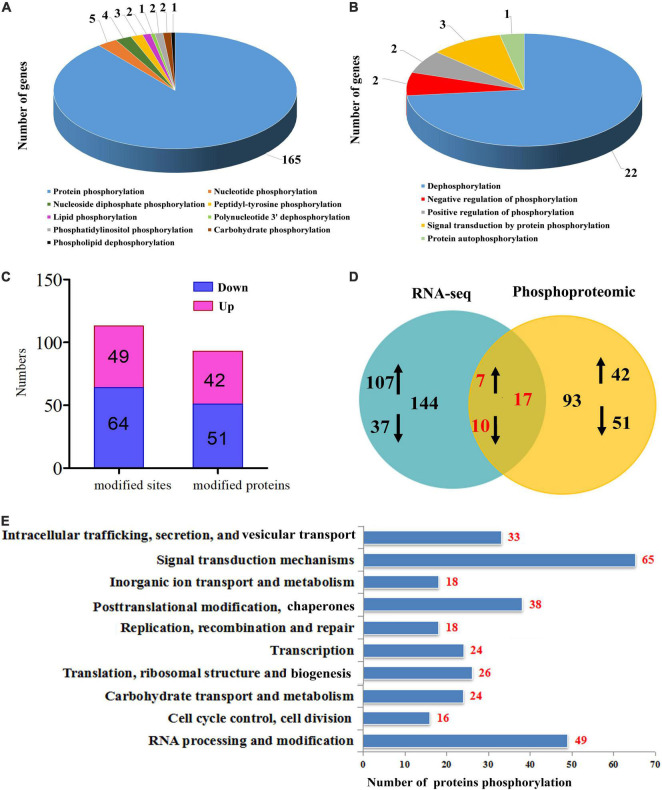
Phosphorylation analysis in tetraploids *Citrus wilsonii* under drought stress. Type of phosphorylation modification **(A)** and modify the model of phosphorylation **(B)** from the RNA-seq data; differentially modified sites (modified proteins) at 2.0 ≤ fold changes or ≤0.5 **(C)**; common and specific differentially phosphorylation modified proteins were compared between RNA-seq data and phosphoproteomic data **(D)**, and the up-regulated gene or protein marked by ↑, down-regulated gene or protein marked by ↓; function classification of phosphorylation modified proteins **(E)**.

Protein phosphorylation was further analyzed through TMT-labeled proteomic analysis of the tetraploid plants under well-watered and water withholding conditions (TD vs TC). Based on the RNA-seq data, 185 genes encoded proteins that could be involved in several kinds of phosphorylation modification. Among these, 165 genes encoded proteins that can be modified by protein phosphorylation in the tetraploids under drought stress (Figure 6A). Phosphorylation includes dephosphorylation, autophosphorylation, and negative or positive regulation of phosphorylation (Figure 6B). The phosphoproteomic analysis identified 113 (2.0 ≤ fold changes or ≤0.5) phosphorylated sites and 93 phosphorylated proteins (2.0 ≤ fold changes or ≤0.5) (Figure 6C). The RNA-seq data showed that 144 genes encoded proteins that could be phosphorylated (2.0 ≤ fold changes or ≤0.5), but only 93 proteins (2.0 ≤ fold changes or ≤0.5) displayed phosphorylation modification using the phosphoproteomic analysis. In addition, only 17 phosphorylated proteins appeared in both the RNA-seq data and the phosphoproteomic analysis data (Figure 6D). We performed functional analysis of these phosphorylated proteins and found that most of these proteins are predicted to participate in signal transduction mechanisms (65), RNA processing and modification (49), and post-translational modification and chaperones (38) (Figure 6E), which correlated with the GO analysis above.

### Kyoto Encyclopedia of Genes and Genomes Annotation

**FIGURE 7:**
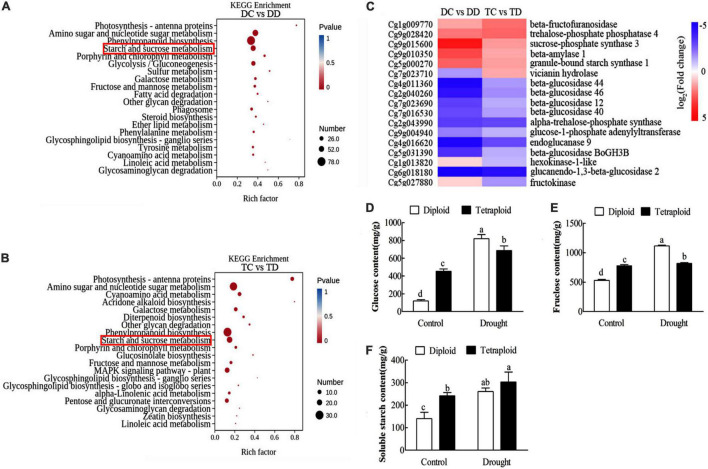
Kyoto Encyclopedia of Genes and Genomes enrichment and hierarchical clustering analysis of the DEGs as well as the physiological analysis between tetraploids and diploids *Citrus wilsonii* in response to drought stress. The top 20 pathways with the highest enrichment level were exhibited according to the amount and enrichment level of DEGs annotated in DC vs DD **(A)** and TC vs TD **(B)**, respectively; hierarchical clustering analysis of the DEGs in starch and sucrose metabolism **(C)**; the contents of glucose **(D)**, fructose **(E)** and soluble starch **(F)** analysis between tetraploids and diploids *Citrus wilsonii* in response to drought stress. Each value is the mean of three biological replicates, and the vertical bars represent the standard errors. Values sharing the same lower case letters are insignificant as ANOVA at *P* < 0.05.

Kyoto Encyclopedia of Genes and Genomes term enrichment was compared between treatments for plants of the same ploidy and between the accessions. The category “phenylpropanoid biosynthesis” contained the greatest number of enriched genes in both the diploids and tetraploids under drought stress (DC vs DD and TC vs TD), followed by “amino sugar and nucleotide sugar metabolism” and “starch and sucrose metabolism” (Figures 7A,B). This revealed that drought stress caused significant changes in both sucrose and secondary metabolism in both diploid and tetraploid plants. The expression patterns of genes in the “starch and sucrose metabolism” pathway were similar in the diploid and tetraploid plants after drought stress (DC vs DD and TC vs TD) (Figure 7C). For example, the genes *beta-fructofuranosidase, sucrose-phosphate synthase 3, beta-amylase1*, and *granule-bound starch synthase1* showed up-regulated expression patterns in both diploid and tetraploid plants after drought stress (DC vs DD and TC vs TD), while the genes *beta-glucosidase 44/46/12/40, alpha-trehalose-phosphate synthase*, and *glucan endo-1,3-beta-glucosidase* showed down-regulated expression patterns in both diploids and tetraploids after drought stress (DC vs DD and TC vs TD) (Figure 7C). To further elucidate the changes in starch and sucrose metabolism, the contents of soluble starch, glucose and fructose were analyzed. The contents of soluble starch, glucose, and fructose significantly increased in both tetraploid and diploid plants in response to drought stress (Figures 7D–F). The data above indicated that tetraploid and diploid Zhique plants showed similar changes in metabolism, particularly sucrose and secondary metabolism, during their response to drought stress.

**FIGURE 8:**
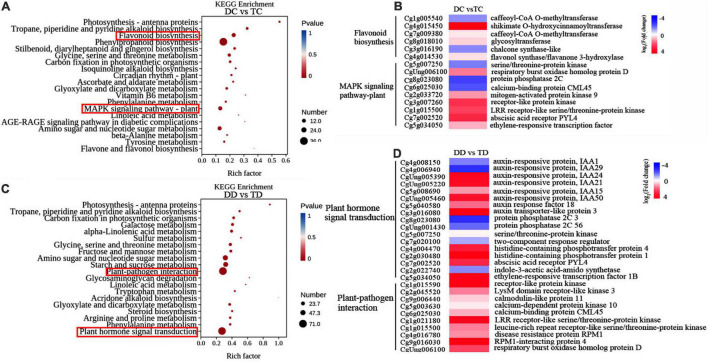
Kyoto Encyclopedia of Genes and Genomes enrichment and hierarchical clustering analysis of the DEGs. The top 20 pathways with the highest enrichment level were exhibited according to the amount and enrichment level of DEGs annotated in DC vs TC **(A)** and DD vs TD **(C)**, respectively; Hierarchical clustering analysis of the DEGs in DC vs TC **(B)** and DD vs TD **(D)**, respectively.

To discern the differences in pathway responses between the diploid and tetraploid plants during drought, we also performed KEGG pathway enrichment analysis of DEGs based on DC vs TC and DD vs TD (Figure 8). Pairwise comparison of the genotypes under well-watered conditions (DC vs TC) revealed that the greatest number of enriched genes were in three pathways, “Phenylpropanoid biosynthesis,” “Flavonoid biosynthesis” and “MAPK signaling pathway” (Figure 8A), indicating that the tetraploid and diploid plants might differ in these metabolic pathways during their development. The expression patterns of several genes related to flavonoid biosynthesis and MAPK signaling were further analyzed using heat map analysis (Figure 8B). Numerous genes related to flavonoid biosynthesis were down-regulated, with the exception of *shikimate O-hydroxycinnamoyl transferase* (Figure 8B). On the contrary, most genes in the MAPK signaling pathway were up-regulated, such as *histidine-containing phosphotransferase protein 1/4*, *receptor-like protein kinase*, *mitogen-activated protein kinase*, *respiratory burst oxidase homolog protein D*, and *LRR receptor-like serine/threonine protein kinase* (Figure 8B). The genes *serine/threonine protein kinase, protein phosphatase 2C, calcium-binding protein CML45*, and *two-component response regulator* were down-regulated in the MAPK signaling pathway (Figure 8B). The greatest number of enriched genes were in the “Plant hormone signal transduction” and “Plant-pathogen interaction” categories in the analysis of DD vs TD (Figure 8C). It is worth noting that almost all analyzed genes in “Plant hormone signal transduction” and “Plant-pathogen interaction” were significantly up-regulated in both the diploids and tetraploids, except *indole-3-acetic acid-amidosynthetase* in the diploid plants (Figure 8D).

### Plant Hormone Levels in Tetraploids and Diploids Under Drought Stress

**FIGURE 9:**
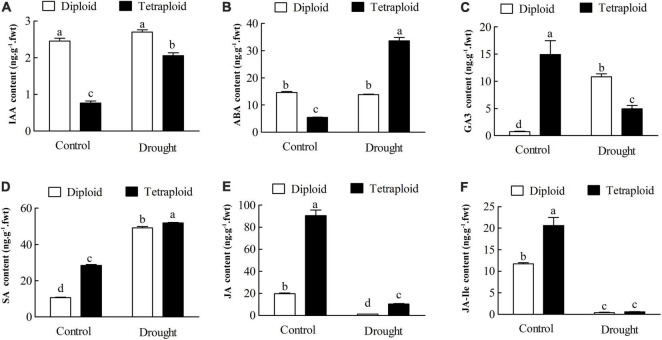
The levels of phytohormone were determined by UHPLC-MS/MS in tetraploids and diploids *Citrus wilsonii* under drought stress. **(A)** IAA content; **(B)** ABA content; **(C)** GA3 content; **(D)** SA content; **(E)** JA content; **(F)** JA-Ile content. Each value is the mean of three biological replicates, and the vertical bars represent the standard errors. Values sharing the same lower case letters are insignificant as ANOVA at *P* < 0.05.

In Drought-Stressed tetraploids, the levels of 3-indoleacetic acid (IAA) and ABA significantly (*P* < *0.05*) increased to 2.69-fold and 6.14-fold higher than the well-watered plants, however, there were no significant (*P* < *0.05*) changes in the diploids compared with the control after drought stress (Figures 9A,B). The content of gibberellin 3 (GA3) in diploids sharply increased by 14.17-fold with drought treatment, butt significantly (*P* < *0.05*) decreased to 33.09% in the drought-stressed tetraploids (Figure 9C). Compared to the well-watered controls, the content of salicylic acid (SA) exhibited a significant (*P* < *0.05*) increase in both diploid (to 4.57-fold) and tetraploid (to 1.82-fold) plants in response to drought stress. In contrast, the levels of jasmonates (JA) and JA-isoleucine (JA-Ile) significantly (*P* < *0.05*) declined in both diploids (to 6.26 and 3.80%) and tetraploids (to 11.53 and 3.03%) with drought stress compared to the control. The results above indicated that tetraploids had different changes in the plant hormone levels from the diploids under drought stress.

## Discussion

Citrus scion varieties are grown on rootstocks to overcome biotic and abiotic problems, thus continued production of citrus requires the discovery of new, stress-tolerant rootstocks (Tadeo et al., 2008). The use of tetraploid rootstock genotypes is an alternative way to improve stress tolerance in many citrus, such as *Carrizo citrange* (Saleh et al., 2008), “Cleopatra” mandarin (Saleh et al., 2008), *C. limonia* (Allario et al., 2013), and *P. trifoliata* (Wei et al., 2019, 2020). In this study, we successfully screened and identified a batch of autotetraploid Zhique seedlings at a frequency of 2.98% in a natural population of seedlings.

### Tetraploidization Caused Greater Tolerance to Drought Stress

In our study, the tetraploids displayed less water loss and cellular damage than the diploids when water was withheld (Figure 2), which indicated that the tetraploid plants have greater tolerance to drought stress. Consistent with our observation, tetraploid *C. limonia* and *P. trifoliata* showed improved water deficit tolerance compared to their corresponding diploids (Allario et al., 2013; Wei et al., 2019). Tetraploid Zhique plants had greener and thicker leaves (Figure 1C) compared to their diploid siblings, which would facilitate leaf water retention and reduce water loss through transpiration. As a matter of fact, the higher chlorophyll content (Figures 5F–H) correlated with the greener leaves in the tetraploids, while larger cells in the leaf (Figures 1J,K) corresponded to the thicker leaves in the tetraploids. Notably, tetraploid Zhique plants also had a lower stomatal density relative to the diploids (Figures 1H,I), which would facilitate reduce transpiration of leaves and was consistent with other results (Wei et al., 2019). In addition, the roots of tetraploid Zhique had fewer branches but were thicker than the corresponding diploids (Figure 1B), and this feature is a useful criterion for initial screening of tetraploids in Zhique. These characteristics may result from the tetraploidization changing the transcript levels of specific genes that then induce phenotypic changes (Tan et al., 2015).

### Tetraploidization Resulted in Higher Photosynthetic Activities Under Drought Stress

It is well established that drought stress influences photosynthesis by closing stomata (Verma et al., 2020; Rios et al., 2021). Our GO enrichment analysis found more DEGs enriched in carbohydrate metabolism in the drought-stressed diploids, however, there were more DEGs enriched in photosynthesis and light harvesting in the tetraploids under drought stress (Figure 4), implying that one of the important reasons for tetraploid adaptation to drought stress might be through the regulation of photosynthesis. Transcriptomic and metabolomic profiling in sesame showed that most enriched drought-responsive genes are mainly related to photosynthesis (You et al., 2019). Photosynthesis is particularly sensitive to drought stress and is often inhibited because of stomatal closure, which reduces CO_2_ supply and furthers metabolic impairment (Hu et al., 2010; Kruse et al., 2019). For example, the declined Gs value indicated that limitations of CO_2_ uptake caused by stomatal closure was responsible for photosynthesis inhibition (Singh et al., 2013). A fast and significant decline in the photosynthetic parameters (P_n_, gs, C_i_, T_r_, and F_v_/F_m_) and chlorophyll content occurred in both diploids and tetraploids under drought stress (Figure 5). Consistent with our results, Shi et al. (2021) reported that photosynthetic parameters (P_n_, gs, C_i_, and T_r_) and water potential were all decreased in *Hordeum jubatum* seedlings under drought stress. In addition, Zhang et al. (2021) reported that drought stress caused declines in the chlorophyll content, P_n_ and Gs values, and F_v_/F_m_ ratio in *Atractylodes lancea*. However, it is worth noting that a lesser decline for these parameters (except T_r_) was observed in tetraploids compared to the diploids, suggesting that tetraploid rootstock maintains higher photosynthetic activities after drought stress in our study (Figure 5). Similarly, Oustric et al. (2017) reported that a higher photosynthetic capacity, including higher P_n_ and Gs values, was observed in tetraploid *C. citrange* rootstocks during cold stress. It is well known that stressors cause chlorophyll breakdown and that reduced chlorophyll content in leaves reduces the photosynthetic capacity (Muller and Munne-Bosch, 2021). In our study, a smaller reduction of chlorophyll content in the tetraploid leaves was observed, which may be another reason for the higher photosynthetic activities in tetraploids under drought stress.

### Phosphorylation Played an Important Role in Tetraploid Tolerance to Drought Stress

Phosphorylation is one of the most important posttranslational protein modifications (PTMs), regulating a wide range of cellular functions, including cell signaling and metabolism as well as stress and defense responses. In this study, various phosphorylation modifications (including dephosphorylation, autophosphorylation, and negative or positive regulation by phosphorylation) occurred in tetraploids subjected to drought stress based on RNA-seq and phosphoproteomics analyses (Figure 6). Phosphorylation is involved in many signaling events and controlled by the activity of a large number of kinases (phosphorylation) and phosphatases (dephosphorylation) (Lenman et al., 2008). These data indicated that tetraploids may switch on phosphorylation modifications by activating protein kinase activity in response to drought stress relative to the diploids. Identification of phosphoproteins and understanding the dynamics of protein phosphorylation, including which residue is phosphorylated or dephosphorylated in response to environmental factors, can help in dissecting the regulatory biological networks at a global level (Kersten et al., 2006). Phosphoproteomics can capture both the dynamics and specificity of protein phosphorylation (Silva-Sanchez et al., 2015). Further analysis of the phosphorylated proteins through TMT-labeled proteomics coupled with IMAC-enrichment analysis identified 113 phosphorylation-modified sites and 93 phosphorylation-modified proteins (Figure 6C). Moreover, these phosphorylated proteins were mainly involved in signal transduction, PTMs, and chaperones (Figure 6E).

### The Changes of Phytohormones Played an Important Role in Tetraploids Tolerance to Drought Stress

Phytohormones play important roles in growth and developmental processes as well as biotic and abiotic stress responses (Verma et al., 2016; Wei et al., 2020). In our KEGG analysis, we found that 255 genes (including 24 DEGs) were enriched in “Plant hormone signal transduction” in tetraploids under drought stress (Figure 8). This was in line with Wei et al. (2020), who showed that the up-regulated DEGs were also enriched in “plant hormone signal transduction” in trifoliate orange tetraploids under salt stress, which included nine DEGs related to auxin, BR, cytokinin, and JA signaling pathways. Among the plant hormones, ABA, SA, and JA are known to play important roles in mediating plant responses to abiotic stresses (Bari and Jones, 2009; Nakashima and Yamaguchi-Shinozaki, 2013). Tetraploid *C. limonia* rootstock increases drought tolerance *via* enhanced constitutive root ABA production (Allario et al., 2013). Our targeted metabolic analysis showed that the levels of IAA and ABA in tetraploids significantly increased with drought stress compared to the watered controls, however, there were no significant changes in the drought-stressed diploids compared with the control (Figures 9A,B). Under drought conditions, ABA is known to stimulate short-term responses like closure of stomata, resulting in maintenance of water balance and longer-term growth responses through regulation of stress-responsive genes (Zhang et al., 2006). It is known that GAs crosstalk with several other hormones, such as IAA, to regulate plant growth and development in response to stresses (Verma et al., 2016). In our study, the content of GA_3_ in diploids sharply increased after drought treatment, on the contrary, GA_3_ significantly (*P* < 0.05) decreased in the tetraploids with drought stress (Figure 9C). Interestingly, SA displayed a significant increase, while JA and JA-Ile exhibited a remarkable decrease in both diploids and tetraploids with drought treatment (Figures 9D–F). Accumulating evidence indicates that SA and JA play major roles in response to biotic stress, as their levels increase with pathogen infection (Bari and Jones, 2009). Surprisingly, a large number genes related to plant pathogen interaction showed differential expression under drought stress (Figure 8), implying that biotic and abiotic stresses may use some common signal transduction mechanisms. The tetraploid rootstock showed different changes in plant hormone signaling than the diploids under drought stress, although the detailed mechanism needs further research.

## Conclusion

In summary, we identified a batch of autotetraploid *C. wilsonii* that displayed stronger drought tolerance. This germplasm will be used as a new rootstock to improve stress tolerance for grafting citrus. The RNA-Seq analysis revealed that a large number of genes involved in photosynthesis response were differentially expressed in tetraploids under drought stress. Our data further demonstrated that tetraploids maintained higher photosynthetic activities than the diploids after drought stress, based on the changes in photosynthetic indices including P_n_, Gs, T_r_, C_i_, and chlorophyll contents. Compared with diploids, phosphorylation was also modified in the tetraploids after drought stress, as detected through TMT-labeled proteomics. These genes or proteins will be further studied by bioinformatics and as potential targets of genetic engineering in the future. In response to drought stress, both tetraploids and diploids activated phenylpropanoid biosynthesis and starch and sucrose metabolism. Additionally, differences in phytohormone accumulation between tetraploids and diploids may also underlie stronger drought tolerance of the tetraploid germplasm. Collectively, our data suggests that synergistic regulation of photosynthesis, phosphorylation and plant hormone accumulation contributes to drought tolerance of autotetraploid *C. wilsonii.* However, these findings derived from potted experiments may need to be further verify in real-world field patterns.

## Data Availability Statement

The datasets presented in this study can be found in online repositories. The names of the repository/repositories and accession number(s) can be found below: The RNA-seq datasets generated for this study can be found in the NCBI SRA accession SRX14238303. All mass spectrometry proteomics data have been deposited to the Proteome Xchange Consortium *via* the PRIDE partner repository with the dataset identifier PXD031236. All supporting data can be found within the article/Supplementary Material.

## Author Contributions

JJ designed the study and wrote the manuscript. NY and LL performed most of the experiments and data analysis. KR performed some experiments. HW and GQ helped in data analysis and presentation. JD and DD provided seeds of Zhique and guidance for cultivation of seedlings. All authors have read and approved the final manuscript.

## Conflict of Interest

The authors declare that the research was conducted in the absence of any commercial or financial relationships that could be construed as a potential conflict of interest.

## Publisher’s Note

All claims expressed in this article are solely those of the authors and do not necessarily represent those of their affiliated organizations, or those of the publisher, the editors and the reviewers. Any product that may be evaluated in this article, or claim that may be made by its manufacturer, is not guaranteed or endorsed by the publisher.

## Footnotes

1
http://cutadapt.readthedocs.io/en/stable/

2
http://citrus.hzau.edu.cn/download.php

3
http://tophat.cbcb.umd.edu/

4
http://www.bioconductor.org/packages/release/bioc/html/DESeq.html

5
http://geneontology.org/

6
http://www.genome.jp/kegg/pathway.html

## Supplementary Material

Supplementary Figure 1 Morphological **(A,B)** and sectional view of fruits and seeds **(C)** in *Citrus wilsonii*.

Supplementary Figure 2 *Citrus wilsonii* autotetraploid seedlings were identified by simple sequence repeat (SSR) genetic analysis. Lanes 1–3 lanes are the diploids and Lanes 4–7 are tetraploids. M: markers. Panels **(a–c)** are results of PCR with three pairs of primers shown in Supplementary Table 6. All 17 tetraploids had been identified by the SSR molecular markers, and four typical band types we only selected for display.

Supplementary Figure 3 Transcript levels of ten selected differentially expressed genes (DEGs), as revealed by RT-qPCR (bars) and RNA-seq (red lines). RT-qPCR data are means ± SE (*n* = 3). FLS2, LRR receptor-like serine/threonine-protein kinase; BMY5, beta-amylase 5; IAA1, auxin-responsive protein IAA1-like; FAD2, delta(12)-fatty-acid desaturase; IFR, isoflavone reductase-like protein; FBPase, fructose-1,6-bisphosphatase; GlcAK1, glucuronokinase 1; CKX1, cytokinin dehydrogenase 1-like; CYP87A3, cytochrome P450 87A3; and PP2C, probable protein phosphatase 2C.

Supplementary Table 1 List of primer sequences used in the identification of autotetraploids seedlings by SSR genetic analysis.

Supplementary Table 2 Differential expression gene primer information of *Citrus wilsonii* by RT-qPCR analysis.

Supplementary Table 3 The detection calibration curves of each plant hormone sample for HPLC determination.

Supplementary Table 4 The data of quality control (QC) and relative standard deviation (RSD) of each plant hormone.

Supplementary Table 5 The raw data of UHPLC-ESI-MS/MS analysis of each plant hormone.

Supplementary Table 6 Summary of RNA-seq data for each sample.

Supplementary Table 7 Summary of alignment rates to the reference genome for each sample.

Supplementary Table 8 DEGs in the leaves from the two samples comparison (DD vs DC).

## Abbreviations

ABA: abscisic acid
ANOVA: analysis of variance
Ci: intercellular CO_2_ concentration
DEGs: differentially expressed genes
ESI: electrospray ionization
FCM: flow cytometry
F_o_: the minimum fluorescence
Fv/Fm: ratio of variable to maximum fluorescence
GA3: gibberellin 3
GO: Gene Ontology
IAA: 3-indoleacetic acid
IMAC: immobilized metal-ion affinity chromatography
JA: jasmonates
KEGG: Kyoto Encyclopedia of Genes and Genomes
MALDI-TOF: matrix-assisted laser desorption/ionization time of flight
MS: mass spectrometry
PCA: principal component analysis
PMF: peptide mass fingerprinting
Pn: net photosynthetic rate
PTMs: posttranslational protein modifications
QC: quality control
qRT-PCR: quantitative real-time PCR
RNA-seq: RNA sequencing
ROS: reactive oxygen species
RSD: relative standard deviation
RWC: relative water content
SA: salicylic acid
SIM: single ion detection
SPSS: statistical product and service solutions
SSR: simple sequence repeat
TCA: trichloroacetic acid
TFA: trifluoroacetic acid
TMT: tandem mass tag
TOF-MS: time-off-flight mass spectrometer
Tr: transpiration rate
gs: stomatal conductance
UPLC: Ultra Performance Liquid Chromatography.
