# Corrigendum: Tetraploidy in *Citrus wilsonii* enhances drought tolerance via synergistic regulation of photosynthesis, phosphorylation, and hormonal changes

**DOI:** 10.3389/fpls.2023.1269493

**Published:** 2023-11-16

**Authors:** Jinglong Jiang, Ni Yang, Li Li, Gongwei Qin, Kexin Ren, Haotian Wang, Jiarui Deng, Dekuan Ding

**Affiliations:** ^1^ School of Biological Science and Engineering, Shaanxi University of Technology, Hanzhong, China; ^2^ Chenggu Fruit Industry Technical Guidance Station, Chenggu, China

**Keywords:** tetraploid, drought, phytohormones, photosynthesis, phosphorylation

In the published article, there was an error in the text. A correction has been made in **Results**, “Tetraploid Zhique Exhibited Enhanced Drought Tolerance”. This sentence previously stated:

“Four-month-old diploid and tetraploid plants were subjected to drought stress by withholding watering for 21 days.”

The corrected sentence appears below:

“Ten-month-old diploid and tetraploid plants were subjected to drought stress by withholding watering for 21 days.”

Two sentences have been added to **Materials and Methods**. The sentences are below:

“The 10-month-old plants employed are not subjected to grafting during the whole experimental process.”

“It is worth noting that there exists a significant difference in the growth status of seedlings cultivated in the open field compared to those in enclosed cultivation rooms.”

A sentence has been added to **Conclusions**. The sentence is below:

“However, these findings derived from potted experiments may need to be further verify in real-world field patterns.”

The authors apologize for these errors and state that this does not change the scientific conclusions of the article in any way. The original article has been updated.

