# Supplementary figures and images for: Tetraploidy in Citrus wilsonii Enhances Drought Tolerance via Synergistic Regulation of Photosynthesis, Phosphorylation, and Hormonal Changes
Source: Front Plant Sci. 2022 Apr 28;13:875011. doi: 10.3389/fpls.2022.875011 (PMC9096895; doi:10.3389/fpls.2022.875011)

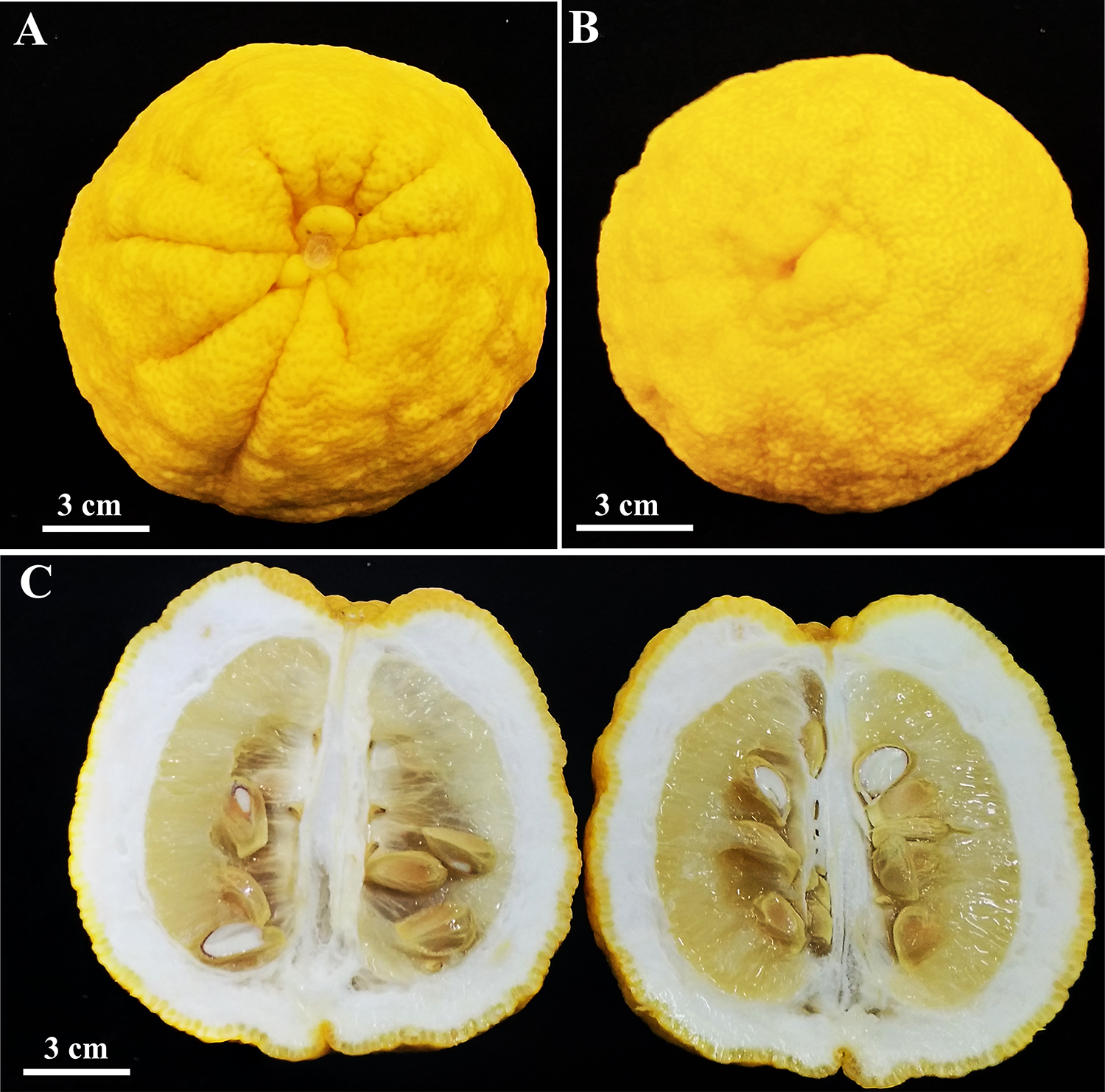

Supplement: Supplementary Figure 1 — Morphological (A,B) and sectional view of fruits and seeds (C) in Citrus wilsonii. [file Image_1.TIF]

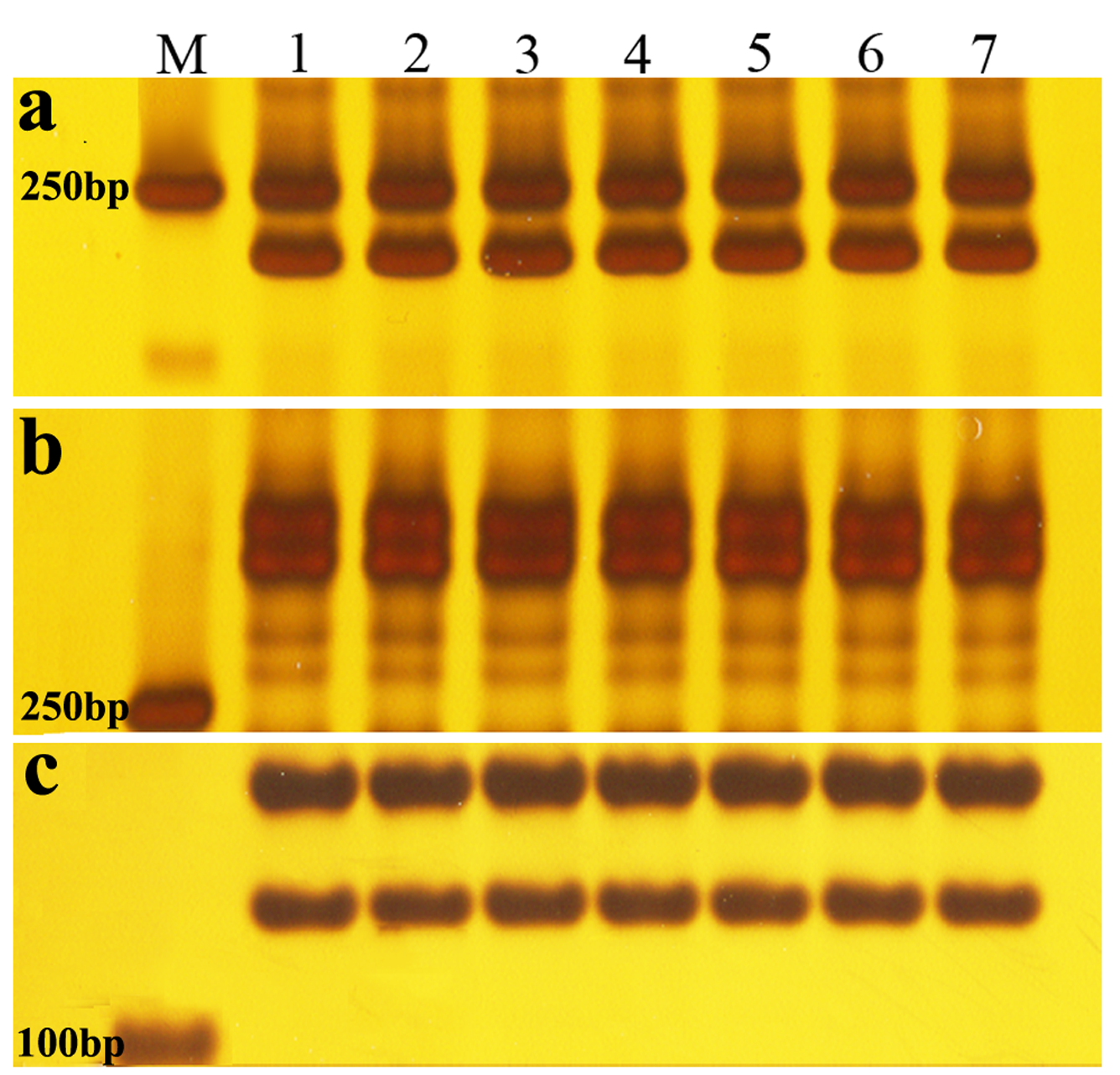

Supplement: Supplementary Figure 2 — Citrus wilsonii autotetraploid seedlings were identified by simple sequence repeat (SSR) genetic analysis. Lanes 1–3 lanes are the diploids and Lanes 4–7 are tetraploids. M: markers. Panels (a–c) are results of PCR with three pairs of primers shown in Supplementary Table 6. All 17 tetraploids had been identified by the SSR molecular markers, and four typical band types we only selected for display. [file Image_2.TIF]

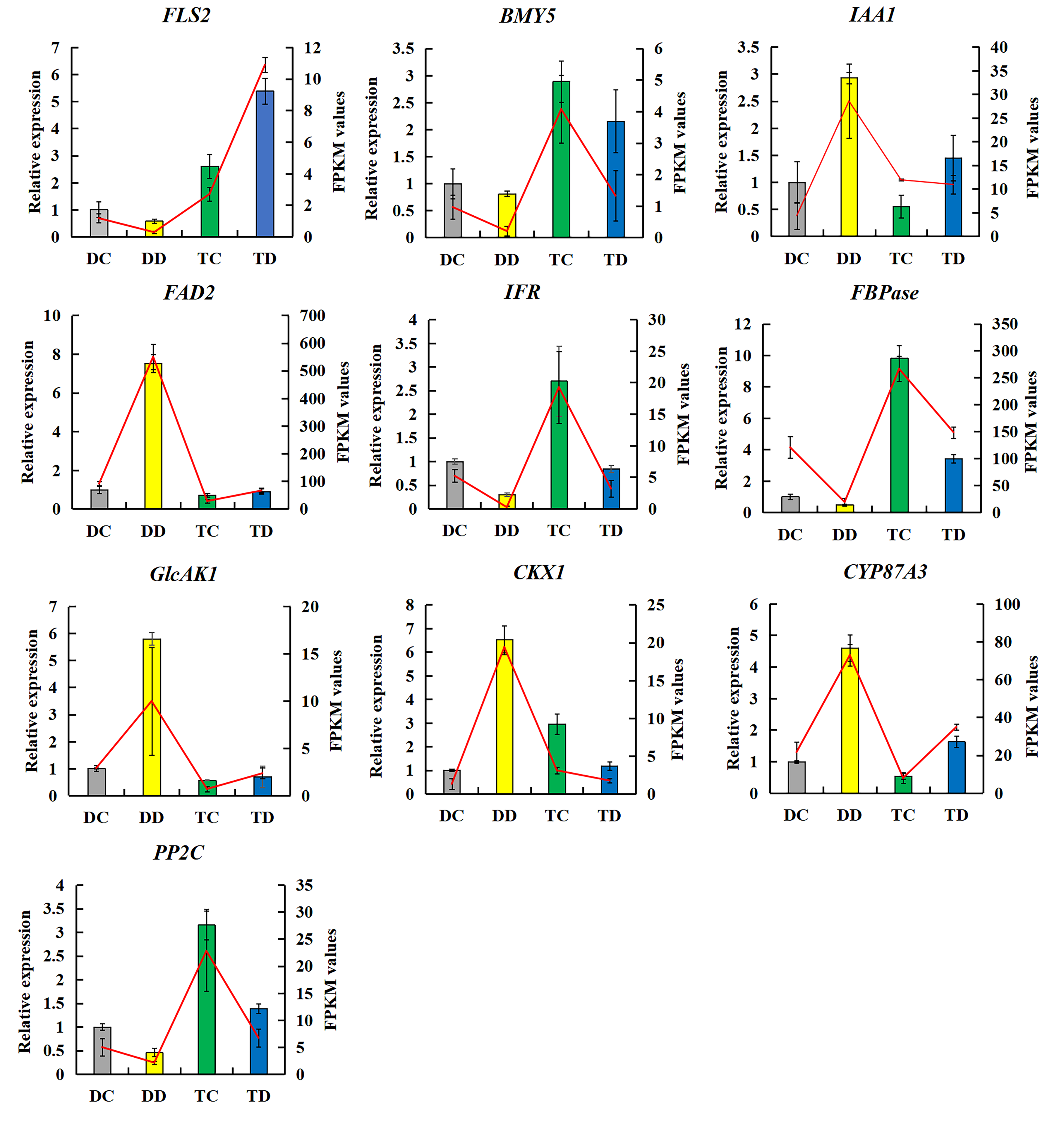

Supplement: Supplementary Figure 3 — Transcript levels of ten selected differentially expressed genes (DEGs), as revealed by RT-qPCR (bars) and RNA-seq (red lines). RT-qPCR data are means ± SE (n = 3). FLS2, LRR receptor-like serine/threonine-protein kinase; BMY5, beta-amylase 5; IAA1, auxin-responsive protein IAA1-like; FAD2, delta(12)-fatty-acid desaturase; IFR, isoflavone reductase-like protein; FBPase, fructose-1,6-bisphosphatase; GlcAK1, glucuronokinase 1; CKX1, cytokinin dehydrogenase 1-like; CYP87A3, cytochrome P450 87A3; and PP2C, probable protein phosphatase 2C. [file Image_3.TIF]
