# Supplementary material for: Tetraploidy in Citrus wilsonii Enhances Drought Tolerance via Synergistic Regulation of Photosynthesis, Phosphorylation, and Hormonal Changes
Source: Front Plant Sci. 2022 Apr 28;13:875011. doi: 10.3389/fpls.2022.875011 (PMC9096895; doi:10.3389/fpls.2022.875011)
Supplement: Supplementary Table 1 — List of primer sequences used in the identification of autotetraploids seedlings by SSR genetic analysis. [file Data_Sheet_1.zip › Supplementary tables(1-8)/Supplementary table S1-7.doc]

**TABLE S1 |** List of primer sequences used in identification of autotetraploids seedlings by SSR genetic analysis

| Primers code | Forward primers (5'-3') | Reverse primers (5'-3') |
| --- | --- | --- |
| a | TCTCACGTCAAAAGACGACG | TCGGCCATAAACCGATACAT |
| b | CGCAAATGACTTCCCAGAAT | GCTCCCTCCGATTCTCTCTC |
| c | GCTCGCAAACACTCTCTGAA | CAAGAAAGGGCAAGAAAACG |

**TABLE S2 |** Differential expression gene primer information of *Citrus wilsonii* by RT-qPCR analysis

| Gene ID | Forward primers (5'-3') | Reverse primers (5'-3') |
| --- | --- | --- |
| Cg1g020830*(FLS2)* | CAACCTGGAACACTCTAAC | GATATTTCAAGCCCGACA |
| Cg2g017760*(BMY5)* | GGTGTGGACAACGAGCCTAT | GAAATTCGCCAATACCAGGA |
| Cg4g008150*(IAA1)* | AGTGGCAGTTGATGGAGCTCCATA | ATATGTAAAACAGTAAAAGGCCTC |
| Cg6g012790*(FAD2)* | ATGGGTGCAGGTGGCAGAATGTCGG | GGTGAGCAGAGCAGCAAAGGTGTA |
| Cg6g023290*(IFR)* | CAGCTGGTCCTCCCAAACAC | GTGGAGGCTTGATGTAGAGGA |
| Cg6g005940*(FBPase)* | GCAGGGGAGACTAACATTCAGGG | CCATTAACACCGCTTCCAGTGC |
| Cg2g007490*(GlcAK1)* | CCTAAACCGGCGATTTCT | ATAAGAAAGAGTGAAGTTTTTGTCA |
| Cg5g004060*(CKX1)* | GATGTTGGGTATGAAGAG | CCTATCATCCCGCTTTTGT |
| Cg5g027670*(CYP87A3 )* | AAGGGCGATGGCTCTGCT | GCAAAGAGCAGCACGAAC |
| Cg9g002100*(PP2C)* | CGCGGACGAGTACAAGAAG | CGCGGCTTCATCATCCA |
| *Actin* | CCGACCGTATGAGCAAGGAAA | TTCCTGTGGACAATGGATGGA |

**TABLE S3 |** The detection calibration curves of each plant hormone samples for HPLC determination.

| Samples of plant hormone | Linear equation | R^2^ |
| --- | --- | --- |
| Gibberellic A3 | Y = 5X + 1.189 | 0.9992 |
| 3-indoleacetic acid | Y = 4X + 7.959 | 0.9994 |
| Salicylic acid | Y = 5X + 6.324 | 0.9987 |
| Abscisic acid | Y = 5X + 1.534 | 0.9995 |
| Jasmonic acid | Y = 5X + 4.913 | 0.9995 |
| Jasmonic Acid-Isoleucine | Y = 5X + 2.336 | 0.9995 |

**TABLE S4 |** The data of quality control (QC) and relative standard deviation (RSD) of each plant hormone.

| Samples of plant hormone | QC1 | QC2 | Average | SD | RSD% |
| --- | --- | --- | --- | --- | --- |
| Gibberellic A3 | 202.78 | 191.981 | 197.3805 | 5.3995 | 2.74 |
| 3-indoleacetic acid | 201.91 | 196.134 | 199.0220 | 2.8880 | 1.45 |
| Salicylic acid | 196.869 | 195.805 | 196.3370 | 0.5320 | 0.27 |
| Abscisic acid | 194.559 | 185.632 | 190.0955 | 4.4635 | 2.35 |
| Jasmonic acid | 198.182 | 191.749 | 194.9655 | 3.2165 | 1.65 |
| Jasmonic acid-isoleucine | 176.987 | 173.314 | 175.1505 | 1.8365 | 1.05 |

**TABLE S5 |** The raw data of UHPLC-ESI-MS/MS analysis of each plant hormone.

| Samples Quality Volume  (mg) (ml) | | | Conversion data (ng/g.FW) | | | | | |
| --- | --- | --- | --- | --- | --- | --- | --- | --- |
|  |  |  | Salicylic acid | Gibberellic A3 | 3-Indoleacetic Acid | Abscisic acid | Jasmonic acid | Jasmonic Acid-Isoleucine |
| DC1 | 99.76 | 0.2 | 10.994 | 0.662 | 2.542 | 14.350 | 19.707 | 11.939 |
| DC2 | 98.92 | 0.2 | 10.590 | 0.853 | 2.408 | 14.430 | 19.337 | 11.438 |
| DC3 | 101.2 | 0.2 | 10.658 | 0.771 | 2.415 | 15.085 | 20.512 | 11.899 |
| DD1 | 100.59 | 0.2 | 49.979 | 11.423 | 2.738 | 14.081 | 1.219 | 0.457 |
| DD2 | 101.44 | 0.2 | 48.226 | 10.193 | 2.737 | 13.681 | 1.205 | 0.406 |
| DD3 | 99.95 | 0.2 | 49.115 | 10.773 | 2.619 | 13.771 | 1.303 | 0.478 |
| TC1 | 98.49 | 0.2 | 28.119 | 12.030 | 0.747 | 5.292 | 85.105 | 18.682 |
| TC2 | 99.82 | 0.2 | 28.968 | 15.901 | 0.719 | 5.570 | 91.743 | 20.886 |
| TC3 | 100.99 | 0.2 | 28.341 | 16.861 | 0.828 | 5.555 | 94.744 | 22.388 |
| TD1 | 99.92 | 0.2 | 52.022 | 4.688 | 1.980 | 33.989 | 10.374 | 0.677 |
| TD2 | 101.34 | 0.2 | 52.062 | 4.515 | 2.074 | 34.626 | 10.866 | 0.657 |
| TD3 | 100.71 | 0.2 | 51.443 | 5.616 | 2.125 | 32.223 | 10.070 | 0.544 |

(DC: diploid under control condition; TC: tetraploid under control condition; DD: diploid under drought stress; TD: tetraploid under drought stress. '1, 2, 3' indicate three repeats.)

**TABLE S6** | Summary of RNA-seq data for each sample.

| **Samples** | | **Raw Reads** | **Clean reads** | **Clean Reads (%)** | **%≥Q20** | **%≥Q30** |
| --- | --- | --- | --- | --- | --- | --- |
| Diploid | DC1 | 53699726 | 49193160 | 91.6 | 97.66 | 93.41 |
|  | DC2 | 44014656 | 40473118 | 91.95 | 97.69 | 93.48 |
|  | DC3 | 47881058 | 43902668 | 91.69 | 97.58 | 93.16 |
|  | DD1 | 57559150 | 52711294 | 91.57 | 97.65 | 93.34 |
|  | DD2 | 46473670 | 42533612 | 91.52 | 97.69 | 93.41 |
|  | DD3 | 47924360 | 43504980 | 90.77 | 97.9 | 93.9 |
| Tetraploid | TC1 | 46033152 | 42011536 | 91.26 | 97.7 | 93.51 |
|  | TC2 | 52575790 | 47651316 | 90.63 | 97.49 | 93.01 |
|  | TC3 | 50083812 | 45686470 | 91.22 | 97.81 | 93.69 |
|  | TD1 | 53916774 | 49552872 | 91.9 | 97.75 | 93.55 |
|  | TD2 | 55304198 | 50842258 | 91.93 | 97.91 | 93.88 |
|  | TD3 | 44522708 | 40757860 | 91.54 | 97.87 | 93.81 |

(DC: diploid under control condition; TC: tetraploid under control condition; DD: diploid under drought stress;

TD: tetraploid under drought stress. '1, 2, 3' indicate three repeats.)

**TABLE S7 |** Summary of alignment rates to the reference genome for each sample.

| **Samples** | | **Clean reads** | **Total Mapped** | **Unique Mapped Reads** | **Multiple Mapped**  **Reads** | **Mapped to Gene** | **Mapped to InterGenic Regions** | **Mapped to Exon** |
| --- | --- | --- | --- | --- | --- | --- | --- | --- |
| Diploid | DC1 | 49193160 | 45267549 (92.02%) | 43916630 (97.02%) | 1350919 (2.98%) | 41524563 (94.55%) | 2392067 (5.45%) | 39910893 (96.11%) |
|  | DC2 | 40473118 | 37194679 (91.90%) | 36127412 (97.13%) | 1067267 (2.87%) | 34129373 (94.47%) | 1998039 (5.53%) | 32746217 (95.95%) |
|  | DC3 | 43902668 | 40091224 (91.32%) | 38917850 (97.07%) | 1173374 (2.93%) | 36561322 (93.94%) | 2356528 (6.06%) | 35007191 (95.75%) |
|  | DD1 | 52711294 | 48371438 (91.77%) | 46942311 (97.05%) | 1429127 (2.95%) | 44387539 (94.56%) | 2554772 (5.44%) | 42172063 (95.01%) |
|  | DD2 | 42533612 | 38463287 (90.43%) | 37200365 (96.72%) | 1262922 (3.28%) | 35166714 (94.53%) | 2033651 (5.47%) | 33544628 (95.39%) |
|  | DD3 | 43504980 | 38854332 (89.31%) | 37545697 (96.63%) | 1308635 (3.37%) | 35540193 (94.66%) | 2005504 (5.34%) | 33958767 (95.55%) |
| Tetraploid | TC1 | 42011536 | 38447183 (91.52%) | 37111160 (96.53%) | 1336023 (3.47%) | 35132851 (94.67%) | 1978309 (5.33%) | 33663258 (95.82%) |
|  | TC2 | 47651316 | 42994728 (90.23%) | 41546637 (96.63%) | 1448091 (3.37%) | 39166099 (94.27%) | 2380538 (5.73%) | 37642380 (96.11%) |
|  | TC3 | 45686470 | 42051430 (92.04%) | 40656466 (96.68%) | 1394964 (3.32%) | 38547952 (94.81%) | 2108514 (5.19%) | 37094888 (96.23%) |
|  | TD1 | 49552872 | 44727139 (90.26%) | 43121785 (96.41%) | 1605354 (3.59%) | 40562774 (94.07%) | 2559011 (5.93%) | 38639608 (95.26%) |
|  | TD2 | 50842258 | 46449889 (91.36%) | 44924084 (96.72%) | 1525805 (3.28%) | 42429167 (94.45%) | 2494917 (5.55%) | 40630160 (95.76%) |
|  | TD3 | 40757860 | 36796653 (90.28%) | 35530675 (96.56%) | 1265978 (3.44%) | 33499660 (94.28%) | 2031015 (5.72%) | 32046150 (95.66%) |

(DC: diploid under control condition; TC: tetraploid under control condition; DD: diploid under drought stress; TD: tetraploid under drought stress. '1, 2, 3' indicate three repeats.)
